# Dual effects of gonadotropin-inhibitory hormone on testicular development in prepubertal Minxinan Black rabbits (*Oryctolagus cuniculus*)

**DOI:** 10.3389/fvets.2024.1320452

**Published:** 2024-01-24

**Authors:** Lei Sang, Shikun Sun, Jinxiang Wang, Chengfang Gao, Dongjin Chen, Xiping Xie

**Affiliations:** Fujian Key Laboratory of Animal Genetics and Breeding, Institute of Animal Husbandry and Veterinary Medicine, Fujian Academy of Agricultural Sciences, Fuzhou, China

**Keywords:** GnIH, dual action, testicular development, spermatogenesis, Minxinan Black rabbit

## Abstract

Gonadotropin-inhibitory hormone (GnIH) is a neurohormone that not only suppresses reproduction at the brain level but also regulates steroidogenesis and gametogenesis at the gonad level. However, its function in gonadal physiology has received little attention in rabbits. The main objective of this study was to evaluate the effects of GnIH on testicular development and function in prepubertal Minxinan Black rabbits (*Oryctolagus cuniculus*). In the present study, we investigated the serum reproductive hormone concentration, testicular parameters, morphology of seminiferous tubules, apoptosis of testicular cells, and expression of reproductive-related genes in male prepubertal Minxinan Black rabbits intraperitoneally administered with 0, 0.5, 5, or 50 μg quail GnIH-related peptides (qGnIH) for 10 days. Compared with the vehicle, administration with 5 μg of qGnIH downregulated the serum testosterone concentration and mRNA levels of spermatogenic genes (*PCNA*, *FSHR, INHβA*, *HSF1*, and *AR*) and upregulated the apoptosis rate of testicular cells; administration with 50 μg of qGnIH decreased the serum testosterone concentration and hypothalamic *GnIH* gene mRNA level and increased the serum LH concentration, pituitary *LHβ* gene mRNA level, testicular weight, gonadosomatic index (GSI), and spermatogenic cell layer thickness. It is concluded that GnIH could exert dual actions on testicular development depending on the male prepubertal rabbits receiving different intraperitoneal doses.

## 1 Introduction

Gonadotropin-inhibiting hormone (GnIH) is a hypothalamic neuropeptide with a C-terminal LPXRFamide (X = L or Q) motif that was originally isolated from the brain of Japanese quail as an inhibitor of gonadotropin-releasing hormone (GtH) (1). An abundance of studies have shown that peptides orthologous to quail GnIH shared identical C-terminal sequences act to control GtH release across vertebrates and are also named GnIH (2–5). Central administration of GnIH reduced gonadotrophin release hormone (GnRH)-stimulated luteinizing hormone β subunit (LHβ) or follicle-stimulating hormone β subunit (*FSHβ*) expression in the pituitary gland and decreased LH and FSH release into the hypophyseal portal systems in sea bass (6), ducks (7), Siberian hamsters (8, 9), and rats (10). Although most studies have shown an inhibitory effect of GnIH on the vertebrate HPG axis, several *in vivo* and *in vitro* studies have shown a stimulatory effect depending on the species, reproductive stages, higher doses or longer duration of GnIH administration (3, 8, 11, 12). For instance, the stimulatory effects of GnIH on GtH synthesis and secretion were reported in European eel (13), *Catla catla* (14), male chub mackerel (15), yellowtail kingfish (12), and hamster (8). Thus, these studies have demonstrated that GnIHs are highly conserved in the regulation of the hypothalamic–pituitary-gonadal (HPG) axis.

GnIH has also been reported to express in gonads and act directly on the gonads mediated by its receptor: G protein-coupled receptor 147 (GPR147) in vertebrates (1, 11, 16). Treatment with GnIH significantly decreased the number of G0/G1 cells and proliferative activity of type A spermatogonia. It inhibited human chorionic gonadotropin (hCG) and FSH-induced spermatogenesis in the *ex-vivo* culture of zebrafish testis (11). The injection of GnIH induced testicular apoptosis, decreased spermatogenic activity and suppressed normal testicular growth in quails (1). Furthermore, GnIH also decreased testicular activity in mice (16), mediated epididymal apoptosis and autophagy in rats (17), and inhibited granulosa cell (GC) proliferation in pigs (18). *In vivo* and *in vitro* studies have shown that GnIH is capable of influencing gonadal development and function.

In prepubertal Minxinan Black rabbits, the testis remains morphologically silent until the animal approaches puberty (approximately 90 days old). Administration of exogenous GnIH might influence pubertal onset and development, resulting in advanced or delayed puberty in rabbits. However, the effects of chronic infusion of GnIH on testicular development and function have never been evaluated in the male prepubertal rabbits. Therefore, in this study, we first examined the effects of GnIH on serum reproductive hormone concentrations and testicular parameters, then evaluated its effects on the morphology of seminiferous tubules and apoptosis rate of testicular cells, and finally detected its effect on the expression of reproductive-related genes in Minxinan Black rabbits aged 90 days.

## 2 Methods

### 2.1 Animals and drugs

In this study, quail GnIH-related peptide 1 (qGnIH) was purchased from Phoenix Pharmaceuticals (catalog No. 040–52, United States), stored at −80°C and dissolved in the vehicle for administration.

Eighty-day-aged Minxinan Black rabbits (*n* = 48 total) weighing 1,500 ± 100 g were used in this study. Based on the previous study (19), three different doses of qGnIH (0.5 μg, 5 μg, 50 μg; *n* = 12 per group) were dissolved in normal saline solution as different treatment groups. Rabbits in the control group (*n* = 12) received vehicle only. Rabbits received intraperitoneal injections of GnIH daily for 10 days. The care and use of rabbits fully complied with local animal welfare laws, guidelines, and policies. The rabbits were allowed free access to food and water at all times and maintained on a 12 h L/12 h D (light/dark) cycle and in an air-conditioned room.

### 2.2 Elisa

Blood samples were collected from the ear vein 24 h after the last administration. After centrifugation (1,000 g, 15 min) of the blood samples, the obtained serum samples were stored at −80°C until further analysis. Serum concentrations of GnRH, FSH, LH and testosterone were measured using ELISA kits following the manufacturer’s instructions (Shanghai Jining Industrial, China).

### 2.3 Measurements and tissue sampling

Rabbits were weighed (live weight defined as LW, kg) prior to sacrifice 24 h after the last administration. The testes were dissected out of the scrotum and the epididymides were removed. Testes were weighed (testicular weight defined as TW, g) and testicular length (defined as L, cm) and width (defined as W, cm) were also measured. Testicular volume (defined as TV, cm^3^) was calculated as TV = 4/3π × (1/2 L) × (1/2 W)^2^ (20) and gonadosomatic index (defined as GSI, g kg^−1^) was calculated as GSI = TW/LW (21, 22). Samples of the hypothalamus, pituitary gland, and left testis were collected and stored in liquid nitrogen until RNA extraction. The right testis was stored in 4% paraformaldehyde for morphometric analysis and TUNEL assay. At the time of tissue collection, none of the rabbits showed signs of disease.

### 2.4 qPCR

Total RNA was isolated from the hypothalamus, pituitary gland, and testis with Trizol™ Reagent (Invitrogen, United States). cDNAs were synthesized using Oligo (dT)_18_ and M-MLV reverse transcriptase (Promega, United States) according to the manufacturer’s instructions. qPCR was performed in triplicate using SYBR Green Master Mix (Applied Biosystems, United States) on a CFX384 Real-time PCR System (Bio-Rad, United States). Glyceraldehyde-3-phosphate dehydrogenase (*GAPDH*) was used as an internal control to normalize the data. The qPCR primers are presented in Table 1.

**Table 1:** Primer information for the genes expressed in the HPG axis.

Gene	Accession No.	Primer sequence (5′ → 3′)	
*GnIH*	MK403675.1	Forward:	GCAGCCACCTTGCCTTTGA
		Reverse:	GTTAGGAATGCGTCTCAAGATGCT
*GnRH*	OP791887	Forward:	GAGAGAGCCAAAGAGGTTGATCAG
		Reverse:	CAGACTTTCCAGAGCTCCTTTCAG
*GnRHR*	NM_001082738.1	Forward:	CACAAATGGATATGGGCAGACAGAA
		Reverse:	CAGCATGATGAGGAGTGGGATA
*ESR1*	XM_008263695.2	Forward:	CCACATCCGCCACATGAGTAAC
		Reverse:	GGTGGGCATCCAGCATCTC
*LHβ*	NM_001082695.1	Forward:	CCCAGTCTGCATCACCTTCAC
		Reverse:	GTCCACGCCAGGTGGACA
*FSHβ*	NM_001082171.2	Forward:	CATCACCATTGCAGTGGAGAAAG
		Reverse:	CACCAGCTCCTTGAAGGTACATA
*AR*	NM_001195724.1	Forward:	GGATGGGACTCATGGTGTTTG
		Reverse:	GGACTTGTGCATACGGTACTCA
*GPR147*	XM_017348647.1	Forward:	CTGGTCACCATCGCGGTCATC
		Reverse:	GGCCTCCCAGCACGAGTAGA
*FSHR*	XM_002709718.2	Forward:	GCCATTGCTGTGCCTTTGC
		Reverse:	CTGCCATAACTGGACTCATCATC
*HSF1*	XM_017340576.1	Forward:	CCCAGCAGCAGAAAGTTGTC
		Reverse:	GCATCAGCGGGATCTTTCTCTT
*HSF2*	XM_002714792.3	Forward:	GATCCTGTAACCATGATGGATTCC
		Reverse:	TCTGGGTCTATGCTAAACTGTCTTC
*LHR*	OL331955	Forward:	CGAAGGTCTCCTCCTCTGAA
		Reverse:	GTTACGGATTCGTTATTCATCCCTTG
*INHβA*	NM_001329069.1	Forward:	GAGAAGGAGCAGTCGCACAGA
		Reverse:	CGATGATCCAGTCATTCCAGCCAAT
*PCNA*	XM_017341762.1	Forward:	GAGATGAATGAGCCAGTGCAACT
		Reverse:	CATACTGAGCGTTACTGTAGGAGA
*GAPDH*	NM_001082253.1	Forward:	CCGCCTGGAGAAAGCTGCTAA
		Reverse:	ACGACCTGGTCCTCGGTGTA

HPG axis, hypothalamic–pituitary-gonadal axis; GnIH, gonadotropin-inhibitory hormone; GnRH, gonadotropin-releasing hormone; GnRHR, GnRH receptor; ESR1, estrogen receptor 1; LHβ, luteinizing hormone beta; FSHβ, follicle-stimulating hormone beta; AR, androgen receptor; GPR147, G protein-coupled receptor 147; FSHR, FSH receptor; HSF1, heat shock factor 1; HSF2, heat shock factor 2; LHR, LH receptor; INHβA, inhibin beta A; PCNA, proliferating cell nuclear antigen; GAPDH, glyceraldehyde-3-phosphate dehydrogenase.

### 2.5 Morphometric analysis

After fixation in 4% paraformaldehyde, testicular tissues were dehydrated with graded 75, 85, 95, and 100% alcohol, infiltrated in xylene, embedded in paraffin blocks, sectioned into 4 μm-thick sections, mounted onto slides, and stained with hematoxylin and eosin (HE). The section images were acquired on a Pannoramic MIDI scanner (3DHISTECH, Hungary). Perimeters and cross-sectional areas of seminiferous tubules (μm and μm^2^) were measured using the liner measurement annotation of Caseviewer (3DHISTECH, Hungary). Thicknesses of spermatogenic cell layers (μm) were measured using liner measurement annotation of Caseviewer (3DHISTECH, Hungary). The thicknesses of each seminiferous tubule were measured 4 times and then averaged. On average, 40–50 tubules were analysed per rabbit.

### 2.6 TUNEL assay

The paraffin-embedded sections (4 μm) were dewaxed with xylene, hydrated using an ethanol gradient, and subsequently subjected to incubation with proteinase K. After performing a TUNEL assay kit containing TdT (Roche Diagnostics, Switzerland) to detect apoptosis-positive cells in rabbit testes, the sections were counterstained with DAPI and mounted onto slides. Fluorescent images were acquired on a Pannoramic MIDI scanner (3DHISTECH, Hungary) using two channels, including DAPI (blue) and TUNEL (green). Cell apoptosis analysis was performed by counting all cells with blue fluorescence (DAPI) and apoptotic cells with green fluorescence (TUNEL) of seminiferous tubules in the sections. The apoptotic rate was calculated as the ratio of the number of apoptotic cells to the total cell count in the testis. AIPATHWELL software (Servicebio, China) for automatic quantification of fluorescent markers was utilized to count the total and apoptotic cells of seminiferous tubules and calculate the apoptosis rate.

### 2.7 Statistical analysis

All data are presented as mean ± SEM. Statistical analysis was performed using one-way ANOVA using SPSS Statistics 17.0 (SPSS Inc., United States). Comparisons between the groups were made by analysing data with the least significant difference (LSD) method. *p*-values of <0.05 were considered to be statistically significant.

## 3 Results

### 3.1 Effects of qGnIH on hormone parameters

The serum concentrations of the reproductive hormones are shown in Table 2. The serum LH level under the high qGnIH-injected concentration (50 μg) was significantly higher than that of the vehicle (*p* < 0.05). Serum testosterone levels under the medium and high qGnIH-injected concentrations (5 and 50 μg) were both significantly lower compared to the vehicle (*p* < 0.05).

**Table 2:** The concentrations of GnRH, FSH, LH, and testosterone in serum.

	Control group (*n* = 12)	0.5 μg group (*n* = 12)	5 μg group (*n* = 12)	50 μg group (*n* = 12)
GnRH (pg/ml)	76.00 ± 10.67	81.02 ± 16.14	103.93 ± 10.65	91.04 ± 4.89
FSH (mIU/ml)	8.99 ± 1.77^ab^	5.79 ± 0.76^a^	11.43 ± 1.49^b^	13.34 ± 1.55^b^
LH (mIU/ml)	14.45 ± 1.23^a^	17.11 ± 1.02^ab^	16.07 ± 0.96^ab^	17.79 ± 1.11^b^
Testosterone (pg/ml)	116.49 ± 7.95^a^	116.11 ± 7.16^a^	80.74 ± 8.93^b^	68.13 ± 6.62^b^

GnRH, gonadotropin-releasing hormone; FSH, follicle-stimulating hormone; LH, luteinizing hormone. Different lowercase alphabets indicate significant differences in different treatment groups (*p* < 0.05).

### 3.2 Effects of qGnIH on testicular parameters

**Figure 1:**
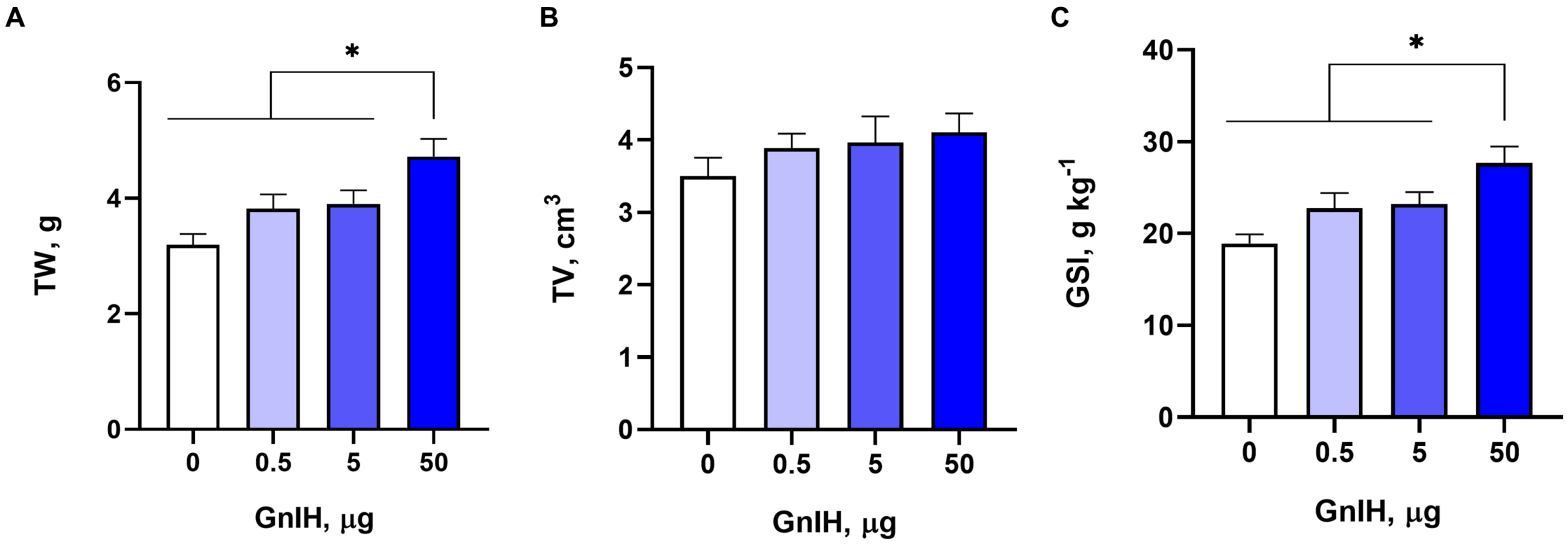
Effects of different injected doses of qGnIH on testicular parameters. **(A)** Effect on testicular weight (TW). **(B)** Effect on testicular volume (TV). **(C)** Effect on gonadosomatic index (GSI). Asterisks indicate significant differences * *p* < 0.05.

To investigate whether qGnIH affects testicular development in male prepubertal rabbits, we measured and calculated the testicular parameters. The TW and GSI were increased by the infusion of 50 μg of qGnIH (*p* < 0.05), while the TV was not changed (*p* > 0.05). The results are shown in Figure 1.

### 3.3 Effects of qGnIH on the morphology of seminiferous tubules

**Figure 2:**
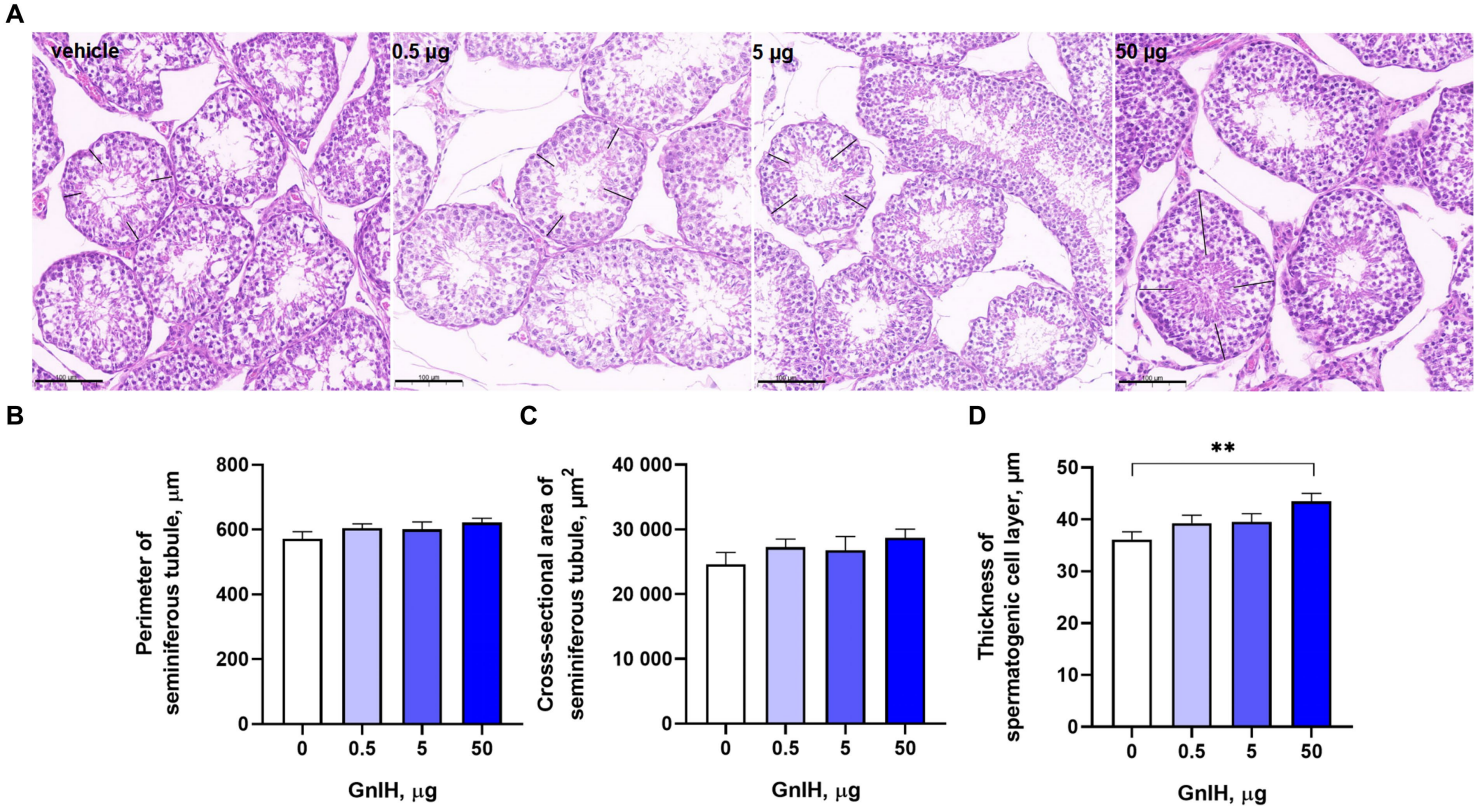
Effects of different injected doses of qGnIH on the morphology of Minxinan Black rabbit seminiferous tubules. **(A)** HE-stained seminiferous tubules (the spermatogenic cell layer thickness marked with the black line segment). Scale bar = 100 μm. **(B)** The perimeter of the seminiferous tubule meaured with the closed polygan annotation of Caseview soft (3DHISTECH, Hungary). **(C)** The cross-sectional area of the seminiferous tubule measured with the closed polygan annotation of Caseview soft (3DHISTECH, Hungary). **(D)** The spermatogenic cell layer thickness of seminiferous tubule measured with the liner measurement annotation of Caseview soft (3DHISTECH, Hungary). Asterisks indicate significant differences ** *p* < 0.01.

The intraperitoneal infusion of qGnIH into prepubertal rabbits had no effect on the perimeters or cross-sectional areas of seminiferous tubules (*p* > 0.05). However, there was a positive effect of 50 μg of qGnIH on the thickness of the spermatogenic cell layer (*p* < 0.01). The results are shown in Figure 2.

### 3.4 Effects of qGnIH on the apoptosis of testicular cells

**Figure 3:**
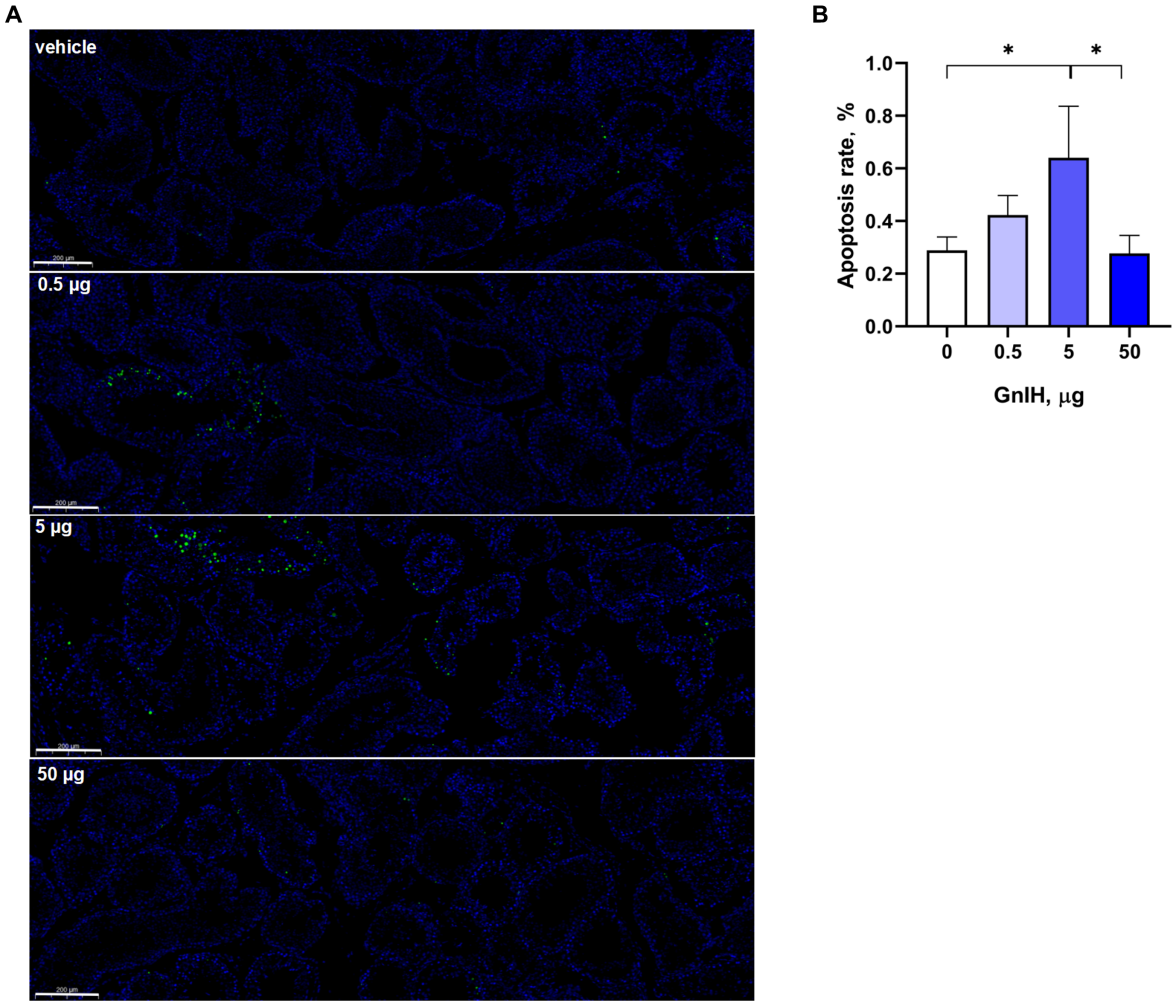
Effects of different injected doses of qGnIH on the apoptosis of Minxinan Black rabbit spermatogenic cells. **(A)** Testicular seminiferous tubules stained with TUNEL and DAPI, green fluorescence represents apoptotic cells (TUNEL staining), and blue fluorescence represents all cells in seminiferous tubules (DAPI staining). Scale bar = 200 μm. **(B)** Apoptotic rate of testicular cells. Asterisks indicate significant differences ^*^ *p* < 0.05.

The apoptosis rates of testicular cells were further detected by TUNEL assay. We observed that infusion of qGnIH at 5 μg increased the apoptosis rate of testicular cells, and neither infusion of GnIH at 0.5 or 50 μg affected the apoptosis rate of testicular cells (*p* > 0.05). Representative images of the TUNEL assay of the apoptosis rate are shown in Figure 3.

### 3.5 Effects of qGnIH on the genes expressed in the HPG axis

#### 3.5.1 Effects of qGnIH on the genes expressed in the hypothalamus

**Figure 4:**
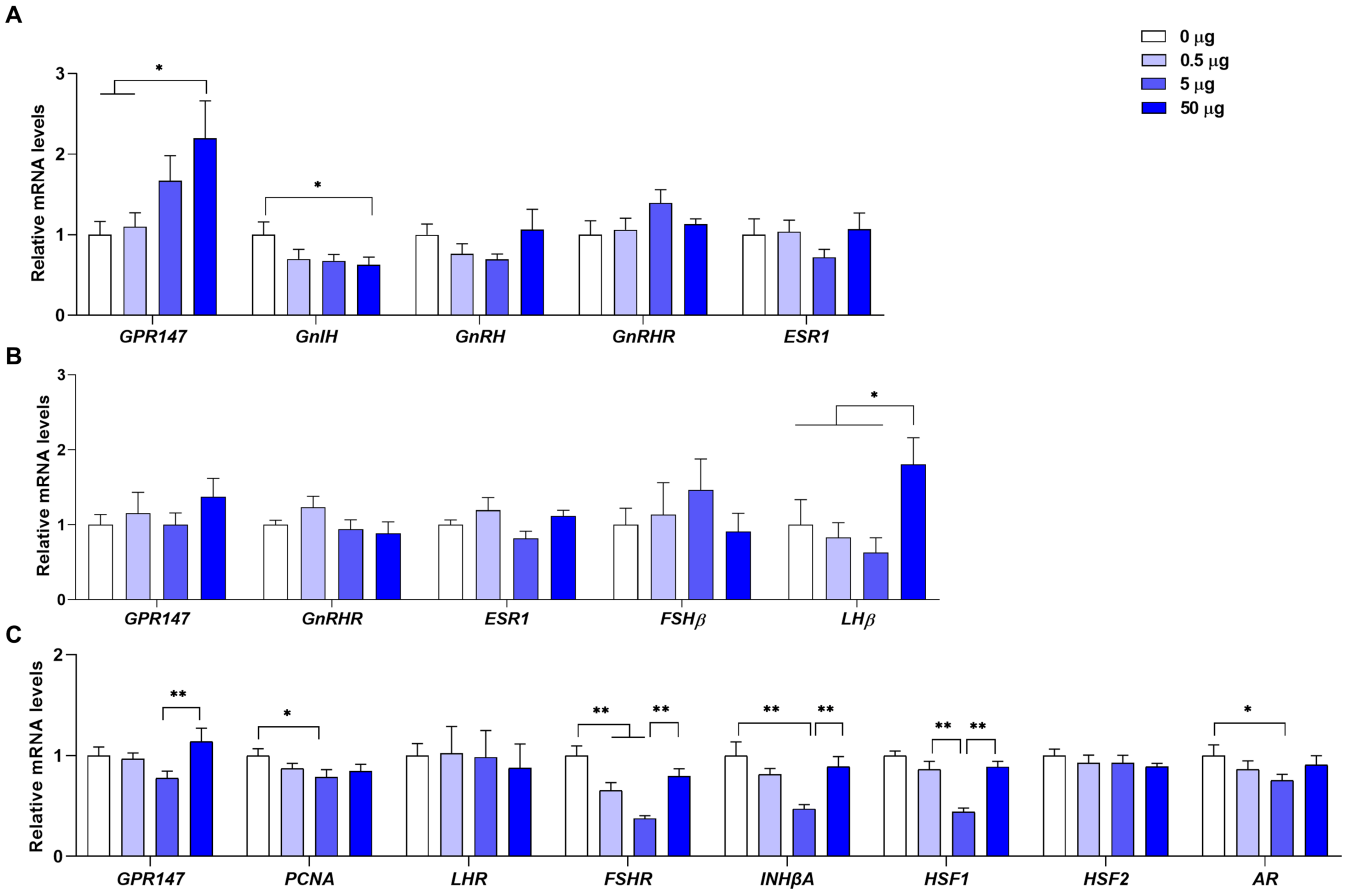
Effects of different injected doses of qGnIH on the mRNA levels of reproductive-related genes in the hypothalamic–pituitary-gonadal (HPG) axis. **(A)** The mRNA levels of reproductive-related genes in the hypothalamus. **(B)** The mRNA levels of reproductive-related genes in the pituitary gland. **(C)** The relative mRNA levels of spermatogenesis genes in testes. Asterisks indicate significant differences ^*^ *p* < 0.05; ^**^ p < 0.01.

As shown in Figure 4A, the mRNA level of *GPR147* was significantly increased (*p* < 0.05) and the mRNA level of *GnIH* gene was significantly decreased (*p* < 0.05) in the hypothalamus at a dose of 50 μg of qGnIH. The mRNA levels of *GnRH*, GnRH receptor (*GnRHR*), and estrogen receptor 1 (*ESR1*) in the hypothalamus were not changed after the injection of qGnIH (*p* > 0.05).

#### 3.5.2 Effects of qGnIH on the genes expressed in the pituitary gland

As shown in Figure 4B, the mRNA level of *LHβ* in the pituitary gland was significantly increased at a dose of 50 μg of qGnIH compared to the vehicle (*p* < 0.05). Moreover, no significant differences were observed for the mRNA levels of *GnRHR*, *FSHβ*, and *ESR* genes after the injection of qGnIH (*p* > 0.05).

#### 3.5.3 Effects of GnIH on the spermatogenic genes expressed in the testes

As shown in Figure 4C, the mRNA levels of proliferating cell nuclear antigen (*PCNA*, *p* < 0.05), FSH receptor (*FSHR*, *p* < 0.01), inhibin beta A (*INHβA*, *p* < 0.01), heat shock factor 1 (*HSF1*, *p* < 0.01), and androgen receptor (*AR*, *p* < 0.05) were lower under the medium qGnIH-injected concentration (5 μg) compared to the vehicle. In addition, although administration with 50 μg of qGnIH did not alter the mRNA levels of testicular spermatogenic genes compared to the vehicle (*p* > 0.05), it increased the mRNA levels of *GPR147* (*p* < 0.01), *FSHR* (*p* < 0.01), *INHβA* (*p* < 0.01), and *HSF1* (*p* < 0.01) compared to administration with 5 μg of qGnIH.

## 4 Discussion

A substantial body of primary research demonstrated that GnIH exerts its regulatory effects on the release or synthesis of GnRH by acting via its receptor-GPR147 in brains. The qGnIH used in the study and rabbit GnIH share a common C-terminal LPXRFamide motif, which plays a crucial role in binding GnIH to its receptor (23). Kriegsfeld et al. reported that the injection of avian GnIH into Syrian hamsters effectively suppressed LH release (24). In the present study, although the administration of 50 μg qGnIH to male rabbits did not alter the hypothalamic mRNA levels of *GnRH*, *GnRHR,* and *ESR1* or the serum *GnRH* level, we also observed that it stimulated *GPR147* expression, suppressed *GnIH* gene expression in the hypothalamus and increased LH synthesis and secretion in the pituitary gland. GnRH is released and transported to the anterior pituitary in an impulsive manner, which affects the synthesis and secretion of downstream hormones (25). Therefore, a single detection of GnRH concentration cannot reveal whether GnIH has the action on GnRH. Taken together, administration of qGnIH could regulate downstream genes in the HPG axis and resultant function via GPR147.

In addition to the distribution in brains of vertebrates, the expression at both the mRNA and protein levels of GnIH and its target receptor gene were also detected in gonads and accessory reproductive organs (26, 27). Moreover, GnIH was also reported to be immunolocalized in the spermatogenic cells, Leydig cells, and interstitial cells of testes in hamsters and pigs throughout sexual development (28, 29). Previous studies demonstrated that gonadal GnIH could directly disrupt the differentiation and maturation of stem cells, as well as the synthesis of sex steroids in gonads of several species in an autocrine or paracrine manner (12, 30). The results of our study indicate that the chronic infusion of qGnIH at a dose of 5 μg into male prepubertal rabbits decreased the concentration of testosterone and the mRNA levels of *PCNA*, *FSHR, INHβA*, *HSF1,* and *AR* genes and increased the resultant apoptosis rate of testicular cells. The expression of *PCNA* is initiated during the period of spermatogonial germ cell proliferation prior to entering meiosis and it regulates spermatogenesis in the control of DNA replication (31). *HSF1* is involved in the reorganization of DNA during spermatid differentiation, and knockout of *HSF1* gene results in meiosis arrest and spermatocyte apoptosis (32). The protein product of the *INHβA* gene, activin A, exerts an influence on germ cell maturation, Sertoli cell function, and the timing of fertility onset (33). Moreover, targeted knockdown of *INHβA* in fetal Leydig cells disrupts testicular cord elongation and expansion, ultimately leading to reduced spermatogenesis and testicular lesions in adulthood (34). *FSHR* involves Sertoli cell proliferation, spermatogenesis, and germ cell survival in testes and the FSH signal transduction pathway via FSHR plays a crucial role in regulating the spermatogenesis in mammalian testes (35, 36). *AR* is involved in the manifestation of secondary sexual characteristics and the initiation and maintenance of spermatogenesis (37). In addition, administration with 5 μg of qGnIH did not alter the concentrations of LH and FSH or the expression levels of *GPR147*, *GnIH* and other related genes expression in brains. Therefore, the findings support the hypothesis that GnIH can directly regulate the secretion of steroid hormones and spermatogenesis in mammalian gonads.

Although the majority of studies conducted in vertebrates have consistently demonstrated the inhibitory effect of GnIH on the HPG axis, several investigations carried out in mammals and fish have revealed its stimulatory action (2, 8, 38). In the present study, the serum LH level and its related gene expression level in the pituitary gland were increased by the intraperitoneal injection of 50 μg qGnIH in prepuberal male rabbits. On the other hand, treatment with qGnIH at 50 μg widened the thickness of the spermatogenic layers, resulting in heavier testicular weight and higher GSI. An electrophysiological study showed that GnIH stimulated 18% of GnRH neurons in proestrus female mice, but no stimulation of GnIH on GnRH neuronal firing was observed in female mice during estrus (39). Moussavi et al. (2) reported that intraperitoneal injection of GnIH in different gonadal stages of goldfish has different effects on the mRNA levels of pituitary *LHβ* and *FSHβ* and serum LH concentration. Peripheral sex-steroid levels of different estrus or gonadal development stages may modify the action of GnIH (38). Moreover, the internalization of GnIH receptors caused by chronic administration or a high concentration of GnIH and the antagonism of injected peptides may lead to the stimulation of GnIH on the HPG axis (38). The central chronic injection of GnIH to Syrian hamsters adapted to short days could increase testicular weight (40). The complex mechanism may be involved in stimulatory and inhibitory effects of GnIH on the HPG axis depending on different exogenous GnIH injected doses and developmental and reproductive stages.

In the present study, the medium dose (5 μg) of qGnIH reduced the serum testosterone level and the expression abundances of spermatogenic genes in the testes but did not alter the synthesis and secretion of reproductive hormone genes in the brain. Thus, at this dose, qGnIH only regulated testicular development and function at the gonadal level, as well as showed inhibitory action. The high dose (50 μg) of qGnIH increased the expression levels of hypothalamic *GPR147* and pituitary *LHβ* genes and the serum LH level, whereas it decreased the serum testosterone level. This suggests that testicular development and function would be dually regulated both by the direct inhibitory action of qGnIH in testes and the stimulatory action of qGnIH-induced LH produced in brains. GnIH may control both GtH release and synthesis in brains to affect the downstream reproductive system (38). In addition, it directly acts on gonadal regulatory steroid hormone production and germ cell differentiation and maturation (38). Moreover, although the high dose (50 μg) of GnIH had no effects on the mRNA levels of spermatogenesis genes and apoptosis rate of testicular cells compared to the control, it increased the mRNA levels of *INHβA*, *HSF1*, and *FSHR* genes and decreased the apoptosis rate of testicular cells compared to the medium dose (5 μg) of GnIH. At a dose of 50 μg of qGnIH, the stimulatory effect of LH may reduce the direct inhibitory action of qGnIH on testicular germ cell differentiation and maturation and the qGnIH still showed a predominant stimulatory effect on testicular development and function in prepubertal male rabbits. Therefore, the heavier testicular weights, the higher GSI and the wider spermatogenic layer thicknesses were observed compared to the control.

## 5 Conclusion

In summary, the present study is the first to investigate the action of GnIH on testicular function and development in prepubertal male Minxinan Black rabbits. The medium and high intraperitoneal administration doses (5 and 50 μg) of qGnIH were found to show dual actions on the regulation of spermatogenesis and exhibit complex regulatory mechanisms in male rabbits. The overall results demonstrate that GnIH may show stimulatory or inhibitory activity on gonadal development and function depending on doses in the male prepubertal rabbits and give a reference to how to employ GnIH in mammals.

## Data availability statement

The original contributions presented in the study are included in the article/supplementary material, further inquiries can be directed to the corresponding author.

## Ethics statement

The animal study was approved by Institutional Animal Care and Use Committee and the Ethics Committee on Animal Experimentation, Fujian Academy of Agricultural Sciences. The study was conducted in accordance with the local legislation and institutional requirements.

## Author contributions

LS: Conceptualization, Funding acquisition, Writing – original draft, Writing – review & editing, Project administration. SS: Project administration, Validation, Writing – review & editing. JW: Formal analysis, Project administration, Writing – review & editing. CG: Project administration, Writing – review & editing. DC: Project administration, Writing – review & editing. XX: Conceptualization, Funding acquisition, Writing – review & editing.
